# TIFFS, TOSSES, AND TURNS: EFFECTS OF AFFECTIVE REACTIVITY TO INTERPERSONAL STRESSORS DURING THE DAY ON NIGHTLY SLEEP

**DOI:** 10.1093/geroni/igad104.1328

**Published:** 2023-12-21

**Authors:** Jin Wen, Patrick Klaiber, Nancy Sin

**Affiliations:** University of British Columbia, Vancouver, British Columbia, Canada; Tilburg University, Tilburg, Noord-Brabant, Netherlands; University of British Columbia, Vancouver, British Columbia, Canada

## Abstract

**Background:**

Sleep has been recognized as an antecedent as well as a consequence of daytime stress. However, less research has compared the role of different stressor types on same-night sleep in the context of daily life. Interpersonal stressors may be particularly important, given that social stressors to elicit greater stress responses than other forms of stressors. This study tested the hypothesis that following days when a person exhibits greater negative affect (NA) reactivity to stressors (versus on days with less NA reactivity), sleep quality will be lower. This link between affective reactivity to stressors and subsequent sleep was expected to be more pronounced for interpersonal stressors versus non-interpersonal stressors.

**Methods:**

In this pre-registered study, 252 adults in British Columbia, Canada (ages 25 to 87y; 68% women; 64% White) completed mobile surveys 5x/day for 14 days to assess daily stressors and NA. Self-reported sleep quality was assessed in morning surveys. Multilevel-models tested daily stressors (interpersonal, non-interpersonal, or no stressors), daily NA (averaged across the day), and their interaction as predictors of subsequent sleep quality, controlling for prior-night sleep quality and sociodemographics.

**Results:**

Daily NA and stressor occurrence independently predicted poorer subsequent sleep quality. Stressor type moderated the relationship between NA and sleep quality, such that this association was stronger for interpersonal compared to non-interpersonal stressors.

**Discussion:**

The findings suggest that encountering interpersonal stressors may be particularly impactful to one’s subsequent sleep. Future studies should consider investigating potential mechanisms that may underlie this association, such as pre-sleep cognitive, emotional, and physiological arousal.

